# Physical Training in Continuation Schools

**Published:** 1921-01-01

**Authors:** 


### Physical Training in Continuation Schools.

### Education Act Opportunities.

Now that the operation of the Education Act is being steadily developed and extended, a good deal of discussion has been going on in the Press with regard to the methods best to be employed in the continuation schools, in which, according to the more enlightened modern outlook, a really valuable work can be done (on right lines) in stimulating the awakened mental faculties of youth at a critical age, and giving them a permanent direction. But the two most important factors necessary to approach the practical ideal are, first, that the continuation teaching shall not be too markedly vocational in character, and, next, that the physical health of the children shall be so cared for as to enable them to regard work in this atmosphere of less compulsion and easier discipline not as a laborious encroachment on their leisure but as interludes in the weekly routine to be looked forward to with pleasure.

### What the System Means.

In this connection too much stress cannot be laid on the value of physical education in these new schools. Sir George Newman, in his recent annual report, made only a casual reference to the matter, though he appeared to recognise its importance, and promised to discuss it in more detail next year, when the scheme of training has progressed further. By physical training one does not mean the old form of "school drill," but an efficient system of education of the body which "encourages the concurrent development of healthy physique, keen intelligence and sound character." The qualities are, as it is necessary to point out over and over again, in a high degree mutually interdependent. Many and various are the forms which such physical training may take, adapted to the needs and capacities of the various ages and possessing the essential elements of freedom, refreshment, elasticity and enjoyment. The Board of Education has rightly emphasised the fact that the new syllabus of physical training (1919) has provided a wide and comprehensive interpretation, including organised physical exercises, games, dancing and swimming. We will add that the Dalcroze system of eurhythmics, described in some detail a few months ago in these columns, provides in itself the highest and most delightful form yet conceived of mental and physical development in combination. Many young women are now being trained specially as exponents of the eurhythmic exercises, and we believe that the time is not far distant when these beautiful movements will be adopted as a matter of course in all our public schools.

### Voluntary Effort Asked For.

It should be remembered that physical training is a compulsory subject in the weekly classes of the continuation schools, and should be of a recreational character. The training should be designed not only to strengthen the body of the growing boy or girl, but with the definite end in view of creating a habit of healthy recreation and open-air pursuits. Somewhere in his report Sir George Newman pointed out that the State has no control over the leisure occupations of the people after school age, and he made an appeal to voluntary bodies interested in physical and social welfare to help to make it possible for young men and women readily to obtain opportunities for games and other forms of recreation. On every hand there is an unprecedented demand for open spaces, fresh air, water and games, etc., for those who spend their days in city offices and factories. There is no reason, he thinks, why this demand should not largely be met if wisely and energetically faced, to the infinite benefit of the physical and moral health of the nation.

### A Yorkshire Example.

The example of a Yorkshire village has been quoted as typical of what can be done in this direction. Early in 1919 a committee was formed to arrange a scheme to provide facilities for recreation for all the inhabitants. Within a year a cricket pitch, six tennis courts and a bowling green had been specially laid; a large room for evening recreation, billiards, dances, etc., had been secured; and, more important still, four football and two hockey grounds had been provided. The opportunity for organised games is now shared by everyone in the village, nearly everyone having become a member of the club; the subscriptions are the same for all classes, and there is a real spirit of sportsmanship and determination to make the scheme a social as well as a recreational success.

It is admitted by every medical officer on the Board of Education, from Sir George Newman downward, and by all who have had long experience of educational problems, that a wisely-applied scheme of physical training is likely to form an integral factor of the success of the continuation schools, as well as for all others in the charge of local education authorities. "It is within their province to ensure that physical education becomes an instrument of the highest national service."

